# Spatial Transcriptomics Analysis Identifies Glial Signatures Spatially Associated with Neuritic Plaques in Human Post‐Mortem Brains

**DOI:** 10.1002/alz.087498

**Published:** 2025-01-03

**Authors:** Berke Karaahmet, Laurine Duquesne, Alina Sigalov, Christina J Yung, Alexandra Kroshilina, Ya Zhang, Mariko Taga, Hans‐Ulrich Klein

**Affiliations:** ^1^ Columbia University Irving Medical Center, New York, NY USA

## Abstract

**Background:**

Glial cells exhibit distinct transcriptional responses to β‐amyloid pathology in Alzheimer’s disease (AD). While sophisticated single‐cell based methods have revealed heterogeneous glial subpopulations in the human AD brain, the histological localization of these multicellular responses to AD pathology has not been fully characterized due to the loss of spatial information. Here, we combined spatial transcriptomics (ST) with immunohistochemistry to explore the molecular mechanisms in the neuritic plaque niche.

**Methods:**

32 sections from the prefrontal cortices of 15 AD and 2 control cases were applied to ST arrays with spatially barcoded probes (spots) of 55µm diameter. Each of the 59,588 tissue‐covering spots in our dataset detected a median of 2,202 genes and included a median of 4 nuclei. Amyloid plaques were stained with Thioflavin S (ThioS) and astrocytes were stained using GFAP in the same ST tissue section. The immunohistochemistry data were then projected onto the ST profiles.

**Results:**

Clustering the spots reconstructed the cortical layer structure. We detected 263 ThioS^+^ amyloid plaques, and spots within 150µm of plaques were compared to distant spots (≥500µm), adjusted for cortical layer and donor. This approach identified 182 plaque‐associated genes and confirmed genes previously reported in mice, such as *GFAP*, *CLU*, *MBP* and *MOBP*. Interestingly, the AD‐related gene *SERPINA3* was found to be upregulated at neuritic plaques. *SERPINA3* is a marker of an inflammatory astrocyte subpopulation, resembling previously discovered disease‐associated astrocytes in mice. Indeed, computationally deconvoluting this astrocyte subpopulation from the ST data confirmed its enrichment at plaques. Further, we found a downregulation of metallothioneins, expressed by astrocytes, indicating a potential dysfunction of metal ion homeostasis. We validate these results using immunohistochemistry and in situ hybridization.

**Conclusions:**

Here, we demonstrate the heterogeneity in glial communities within the amyloid plaque microenvironment. We show evidence suggesting a spatial enrichment of SERPINA3^+^ astrocytes at neuritic plaques. Furthermore, the identification of the 182 plaque‐proximal genes suggests involvement of processes related to inflammation, and ion transport and homeostasis. The role of these subtypes and molecular signatures in AD pathophysiology remains to be elucidated.

